# *Apo* state pore opening as functional basis of increased EAAT anion channel activity in episodic ataxia 6

**DOI:** 10.3389/fphys.2023.1147216

**Published:** 2023-07-19

**Authors:** Mariia Suslova, Daniel Kortzak, Jan-Philipp Machtens, Peter Kovermann, Christoph Fahlke

**Affiliations:** Institute of Biological Information Processing, Molekular-und Zellphysiologie (IBI-1), Forschungszentrum Jülich, Jülich, Germany

**Keywords:** neurological diseases, epilepsy, ataxia, glutamate transport, anion channel, kinetic modeling

## Abstract

*SLC1A2* and *SLC1A3* encode the glial glutamate transporters EAAT2 and EAAT1, which are not only the predominant glutamate uptake carriers in our brain, but also function as anion channels. Two homologous mutations, which predict substitutions of prolines in the center of the fifth transmembrane helix by arginine (P289R EAAT2, P290R EAAT1), have been identified in patients with epileptic encephalopathy (*SLC1A2*) or with episodic ataxia type 6 (*SLC1A3*). Both mutations have been shown to impair glutamate uptake and to increase anion conduction. The molecular processes that link the disease-causing mutations to two major alterations of glutamate transporter function remain insufficiently understood. The mutated proline is conserved in every EAAT. Since the pathogenic changes mainly affect the anion channel function, we here study the functional consequences of the homologous P312R mutation in the neuronal glutamate transporter EAAT4, a low capacity glutamate transporter with predominant anion channel function. To assess the impact of charge and structure of the inserted amino acid for the observed functional changes, we generated and functionally evaluated not only P312R, but also substitutions of P312 with all other amino acids. However, only exchange of proline by arginine, lysine, histidine and asparagine were functionally tolerated. We compared WT, P312R and P312N EAAT4 using a combination of cellular electrophysiology, fast substrate application and kinetic modelling. We found that WT and mutant EAAT4 anion currents can be described with a 11-state model of the transport cycle, in which several states are connected to branching anion channel states to account for the EAAT anion channel function. Substitutions of P312 modify various transitions describing substrate binding/unbinding, translocation or anion channel opening. Most importantly, P312R generates a new anion conducting state that is accessible in the outward facing *apo* state and that is the main determinant of the increased anion conduction of EAAT transporters carrying this mutation. Our work provides a quantitative description how a naturally occurring mutation changes glutamate uptake and anion currents in two genetic diseases.

## Introduction

Glutamate is the major excitatory neurotransmitter in the mammalian brain. After release from the presynaptic nerve terminal excitatory amino acid transporters (EAATs) quickly take up glutamate into surrounding glial and neuronal cells (Rose et al., 2018). EAAT-mediated glutamate transport increases the temporal resolution of glutamatergic synaptic transmission and reduces resting glutamate concentrations. In addition to secondary active glutamate transport, the EAATs also function as glutamate-gated anion channels (Wadiche et al., 1995; Larsson et al., 1996; Machtens et al., 2015). Various cellular functions have been suggested, but the physiological importance of the EAAT anion channel function remains insufficiently understood (Untiet et al., 2017; Engels et al., 2021; Gehlen et al., 2021; Kovermann et al., 2022a).

Impaired EAAT functions have been associated with several human genetic diseases. The first EAATopathy was a genetic condition combining ataxia, hemiplegia, and seizure, called episodic ataxia type 6 (EA6), and shown to be caused by mutations in *SLC1A3*, the gene encoding the glial glutamate transporter EAAT1 (Jen et al., 2005; de Vries et al., 2009; Pyle et al., 2015; Choi et al., 2017a; Choi et al., 2017b; Iwama et al., 2017; Chivukula et al., 2020). Later, mutations in *SLC1* genes were linked to other neurological conditions such as migraine or epilepsy (Epi4K Consortium, 2016; Guella et al., 2017; Kovermann et al., 2017; Stergachis et al., 2019), as well as to psychiatric conditions such as ADHD or Tourette’ syndrome (Adamczyk et al., 2011; van Amen-Hellebrekers et al., 2016).

**FIGURE 1:**
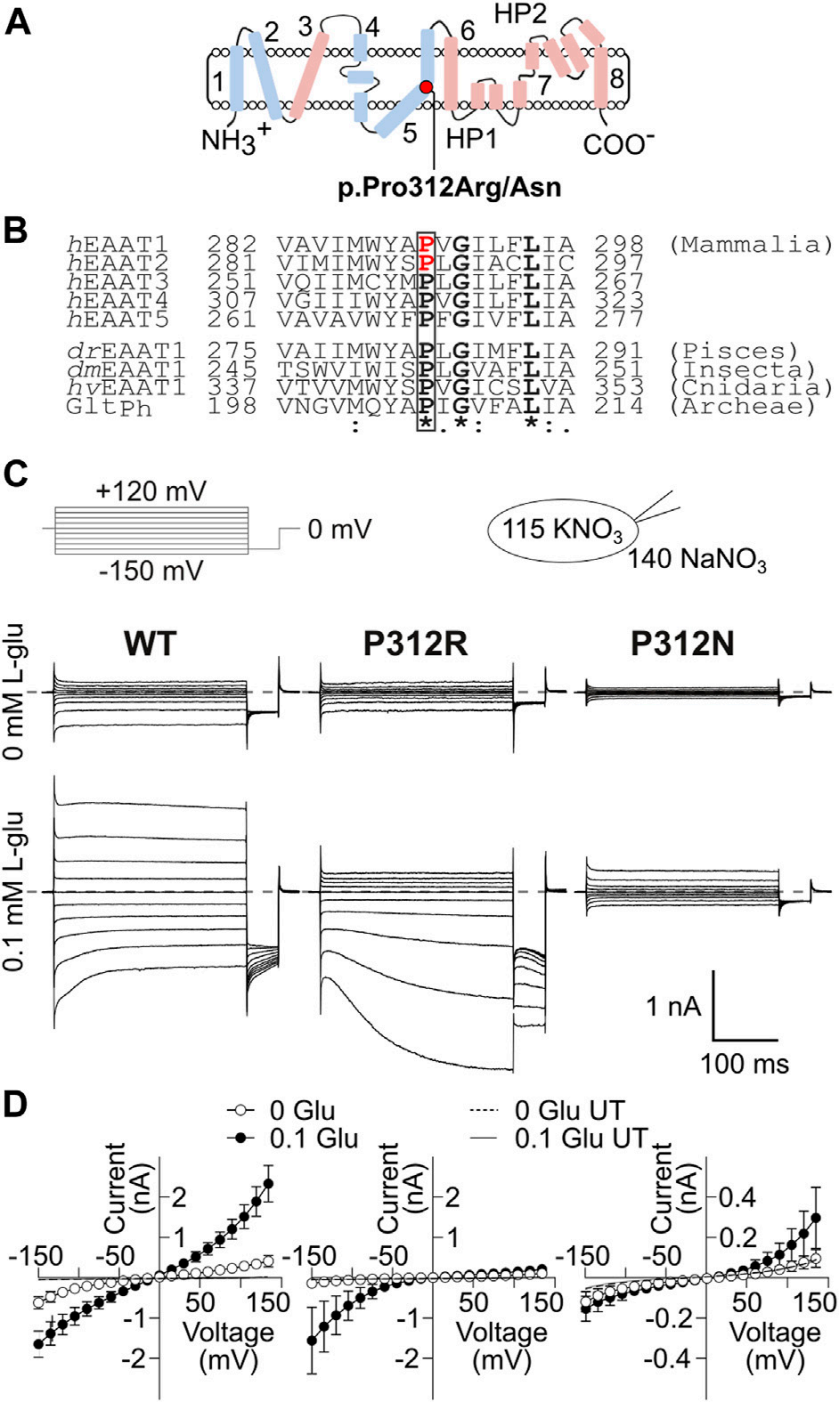
Mutations in P312 modify the time, voltage and substrate dependence of EAAT4 anion currents. **(A)** Position of P312 in an EAAT transmembrane topology model (trimerization/transport domains: blue/magenta, left panel). **(B)** Sequence alignments of mammalian EAAT isoforms and homologues from animals and bacteria illustrate the strict evolutionary conservation of a **PXGXXXL** motif in this class of transporters. Positions of missense mutations associated to human neurological diseases are indicated in red (Jen et al., 2005; Guella et al., 2017; h: *H. sapiens*, dr: *D. rerio*, dm: *D. melanogaster*, hv: *H. vulgaris*, Ph: Pyrococcus horikoshii). **(C)** Representative current recordings from HEK293T cells expressing WT (left), P312R (middle) or P312N (right) *r*EAAT4 internally dialyzed with a KNO_3_-based solution and a NaNO_3_-based external solution before (upper row) and after application of 0.1 mM L-glutamate (lower row). Zero current level is shown as grey dashed line. **(D)** Voltage dependence of mean anion current amplitudes from cells expressing WT (left), P312R (middle) or P312N (right) EAAT4 in the presence as well as in the absence of glutamate. Background currents obtained with untransfected HEK293T cells are shown as solid line in the presence of glutamate and dashed line in the absence of it. Data are given as the means ± 95% *C.I*.; *n* ≥ 9.

Jen et al. (2005) reported a patient with episodic ataxia 6, who was heterozygous for a missense mutation causing the substitution of a highly conserved proline at position 290 by arginine (Figure 1A). This mutation affects both transport functions of EAAT1 in opposite ways; it reduces glutamate transport rates, but increases the EAAT1 anion conductance (Winter et al., 2012; Hotzy et al., 2013). In a transgenic animal model, the *Slc1a3* ^*P290R/+*^mouse, increased EAAT1/GLAST-mediated chloride efflux causes apoptosis of Bergmann glia in early infancy, resulting in cerebellar dysfunction via impaired glutamate reuptake and cerebellar network formation (Kovermann et al., 2020). Transgenic *Drosophila* models of this case of episodic ataxia also demonstrated pathogenic changes in EAAT anion channel function (Parinejad et al., 2016; Wu et al., 2022). Recently, the homologous mutation in *SLC1A2*, predicting P289R EAAT2, was found in patients with early-onset epilepsy and severe developmental delay (Guella et al., 2017) and was also shown to cause increased activity of EAAT2 anion channels (Kovermann et al., 2022b).

Both mutations affect a proline that is conserved in every SLC1 transporter as well as in prokaryotic and eukaryotic homologues (Figure 1B). Since the P290R/P289R mutations cause disease symptoms mainly via gain-of-anion channel function, we decided to study the mechanistic basis of this dysfunction in a related transporter that predominantly functions as glutamate-gated anion channel, in EAAT4 (Fairman et al., 1995; Melzer et al., 2003; Mim et al., 2005). We systematically evaluated the effects of substitutions of the homologous proline with various amino acids. EAAT4 has been studied extensively by our group and was shown to functionally tolerate various point mutations (Kovermann et al., 2010; Machtens et al., 2015). We expressed WT and mutant EAAT4 in mammalian cells and studied transport and anion currents. Time, substrate and voltage dependences of such currents were described with a kinetic model to identify conformational changes that are affected by those mutations.

## Materials and methods

### Heterologous expression of WT and mutant EAAT4 in mammalian cells

pcDNA3.1(−) *r*EAAT4 was kindly provided by Dr. J. Rothstein, Johns Hopkins University, Baltimore, MD, United States, and modified by linking the coding region of monomeric yellow fluorescent protein (mYFP) to the 5´ end of the EAAT4 coding region using PCR-based strategies (Nothmann et al., 2011). Point mutations were generated by PCR-based techniques as described (Leinenweber et al., 2011). For transient transfection of HEK293T cells, we used the Ca_3_(PO_4_)_2_ technique as described (Hebeisen and Fahlke, 2005). For WT and mutant EAAT4-mYFP, two independent recombinants were examined, without obvious functional differences.

### Electrophysiology

Standard whole-cell patch-clamp recordings were performed using an Axopatch 200B amplifier (Molecular Devices, San Jose, United States), with borosilicate pipettes with resistances between 1.0 to 2.0 MΩ (Garcia-Olivares et al., 2008). To reduce voltage errors, more than 80% of the series resistance was compensated by an analog procedure, and cells with current amplitudes >7 nA were excluded from analysis. Currents were filtered at 5 or 10 kHz and digitized with sampling rates of 50 kHz using a Digidata 1322A AD/DA converter (Molecular Devices, San Jose, United States). Cells were clamped to 0 mV for at least 2 s between two test sweeps. In experiments to study EAAT4 anion currents internal solutions contained either (in mM) 115 KNO_3_, 2 MgCl_2_, 5 EGTA, 10 HEPES, pH 7.4; or 115 NaNO_3_, 2 MgCl_2_, 5 EGTA, 10 HEPES, ±0.1 L-glutamate, pH 7.4. Bath solution contained: 140 NaNO_3_ or CholineNO_3_, 5 TEA-Cl, 4 KCl, 2 CaCl_2_, 1 MgCl_2_, 5 HEPES, ±0.1 L-glutamate, pH 7.4. Anion currents were determined without subtraction procedure. For rapid solution exchange to solutions, in which 140 NaNO_3_ was substituted with KNO_3_, or 140 CholineNO_3_ by 140 NaNO_3_ + 1 L-glutamate or in which 0.1 L-glutamate was added to 140 NaNO_3_, a self-assembled piezo-driven system with a dual-channel theta glass tubing was used (Carbone and Plested, 2012). After pulling, pipette tips were reheated and bended to permit horizontal solution flow out of the mounted pipette and subsequently briefly incubated in fluoric acid to thin the theta tube filament. To decrease pipette vibration, a manually smoothed pulse was fed to the piezo amplifier. The speed of the solution exchange–estimated as 10%–90% rise times of open pipette responses–was 658 ± 53 µs (*n* = 56).

Glutamate transport currents were measured in cells dialyzed with (in mM) 115 K-D-gluconate, 2 Mg-D-gluconate_2_, 5 EGTA, 10 HEPES, pH 7.4. Cells were subsequently perfused with solutions containing 140 Na-D-gluconate, 5 TEA-D-gluconate, 4 K-D-gluconate, 2 Ca-D-gluconate_2_, 1 Mg-D-gluconate_2_, 5 HEPES, pH 7.4, with or without 0.1 L-glutamate. Transport currents were determined as L-glutamate-sensitive currents by subtracting currents obtained in the absence of glutamate from currents measured in the presence of glutamate. For all experiments, Ag/AgCl electrodes were connected via external and/or internal agar salt bridges, made from plastic tubing filled with 3 M KCl in 1% agar. Offset potentials were determined at the end of each experiment, and junction potentials were corrected using the JPCalc software (Dr. P. Barry, University of South Wales, Sydney, Australia).

### Confocal microscopy and biochemistry

HEK293T cells were plated on poly-L-lysine coated coverslips 48 h after cell transfection and imaged with an inverted microscope (LSM 780, Carl Zeiss, Jena, Germany) using a 63*x*/1.40 NA oil immersion objective. mYFP was excited at 488 nm (argon laser), and emission was imaged between 543–549 nm, and fluorescences were analyzed with Fiji image analysis software (NIH). Transfection rates were quantified as ratios of transfected cells by the total number of cells (*n* = 1736/1596/788 cells; WT/P312R/P312N) with a 20*x*/1.4 NA oil immersion objective for three independent cell transfections for each construct.

For SDS-PAGEs, HEK293T cells were harvested 18 h after transfection with EAAT4-mYFP fusion proteins and lysed in phosphate buffer, supplemented with 0.4% dodecyl-maltoside for 30′ on ice. Lysates were then centrifuged for 35′ (13,000 rpm, 4°C) and equal amounts of whole cell lysates were analyzed with SDS-PAGEs (12%) and fluorescence scanning (Typhoon FLA 9500, GE Healthcare Europe GmbH, Freiburg, Germany). Each variant (WT/P312R/P312N) was tested in three independent transfections. Complex glycosylation was tested with recombinant PNGase F (New England Biolabs, Ipswich, MA, United States) at a concentration of 16.6 U/μL sample volume for 30´ at 37°C.

### Kinetic modeling

Simulations of EAAT4 anion channel open probabilities were performed by solving differential equations on the basis of a modified EAAT2/Glt-1 model (Bergles et al., 2002; Machtens et al., 2011; Kovermann et al., 2017). Transitions within this kinetic scheme (Figure 7) were estimated by fitting the model predictions to experimentally determined current responses upon rapid substrate applications/removals using a genetic algorithm for minimization of squared errors as implemented in the Python package DEAP (Fortin et al., 2012). Starting values of rate constants were arbitrarily set to 1000 and for gating charges to 0.5. Parameters were “mutated” by adding a random number sampled from a normal distribution with zero mean. The size of these changes are controlled by the standard deviation of this distribution, which we adjusted over the course of fitting. Fitting started with large steps during an initial exploration phase, and steps were later restricted to 10% of the parameter value during refinement.

We initially calculated the steady-state distribution of all transporter states for each conditions before substrate application; these values subsequently serve as initial values to numerically solve a linear system of differential equations and to provide time and voltage-dependent absolute open probability $p\left(t,V\right)$ . Absolute open probabilities were converted into macroscopic currents by adding occupancies of each channel state assuming that all open-channel states exhibit same unitary conductance (Kovermann et al., 2010).

$$I\left(t,V\right)=N∙p\left(t,V\right)∙i\left(V\right)$$

with N being the number of channels in the membrane, *t* the time, *V* the voltage, *i(V)* the single channel amplitude and *p(V)* the absolute open probability. Dividing the current time course by current amplitudes at the end of the application provides

$$I_{norm1}\left(t,V\right)=\frac{I\left(t,V\right)}{I\left(t_{end},V\right)}=\frac{p\left(t,V\right)}{p\left(t_{end},V\right)}$$

Assuming a constant single-channel conductance (Kovermann et al., 2010), the normalization of currents to steady-state current amplitude (*I*_*SS*_) at −150 mV *(V_0_)* provides the voltage dependence of $p\left(t,V\right)$

$$I\left(t,V\right)=N∙p\left(t,V\right)∙i\left(V\right)$$

$$i\left(V\right)=\gamma ∙V$$

$$I_{norm2}\left(t,V\right)=\frac{I\left(t,V\right)}{I\left(t_{end}{,V}_{0}\right)}=\frac{p\left(t,V\right)}{p\left(t_{end},V_{0}\right)}∙\frac{V}{V_{0}}$$

Our fitting procedure aimed at minimizing the sum of squared residuals (*SSR1*) between experimentally determined $I_{norm1}\left(t,V\right)$ and simulated $\frac{p\left(t,V\right)}{p\left(t_{end},V\right)}$

$$SSR1=\sum {\left(I_{norm1}\left(t,V\right)-\frac{p\left(t,V\right)}{p\left(t_{end},V\right)}\right)}^{2}$$

and SSR2 between $I_{norm2}\left(t,V\right)$ and simulated $\frac{p\left(t,V\right)}{p\left(t_{end},V_{0}\right)}∙\frac{V}{V_{0}}$

$$SSR2=\sum {\left(I_{norm2}\left(t,V\right)-\frac{p\left(t,V\right)}{p\left(t_{end},V_{0}\right)}\frac{V}{V_{0}}\right)}^{2}$$

WT EAAT4 anion channels exhibit unitary conductances with negligible voltage dependence (Kovermann et al., 2010). We do not know how the mutations affect this unitary conductance. We therefore weighted *SSR2* lower in mutants than in WT and allowed 20% deviation from linearity.

For plotting and error calculation, experimental currents were normalized as in *I_norm2_* and plotted together with simulated traces (Figure 7). Experimental and predicted values were normalized to 1 at the end of the application of the most negative voltage. We quantified the overall error function or goodness of fit as the sum of all the individual SSRs from the different application experiments.

After optimizing fit parameters, we used a modified genetic algorithm to estimate the range of fit parameters with comparable quality of fit, as a value equivalent to a statistical error of the fit parameters (Figure 9). For this, fit parameters were randomly modified in 3000 generations of an explorative genetic algorithm to collect parameter values that impaired the goodness of fit by less than 25%.

### Data analysis

Data were analyzed using a combination of Clampfit (Molecular Devices, San Jose, United States), Origin (OriginLab Corp., Northampton, MA, United States), SigmaPlot (Systat Software GmbH, Düsseldorf, Germany), Excel (Microsoft Corp., Redmont, WA, United States) and Libre office Calc (The Document Foundation, Berlin, Germany) software. All summary data are given as means ± *C.I.* (95%-confidence interval) or as box-whisker plots drawn between the first and third quartiles with whiskers providing the upper and lower 95% of the data range. Fluorescence levels of confocal images were analyzed with 2-way ANOVA with WT as controls, and Holm-Sidak *post hoc* testing, protein amounts and transfection rates were compared with one-way ANOVA, and Holm-Sidak *post hoc* testing. For comparison of dissociation constants and glutamate transport values, Kruskal–Wallis and Dunn’s *post hoc* tests were used.

## Results

### EAAT4 functionally tolerates only few P312 substitutions

**FIGURE 2:**
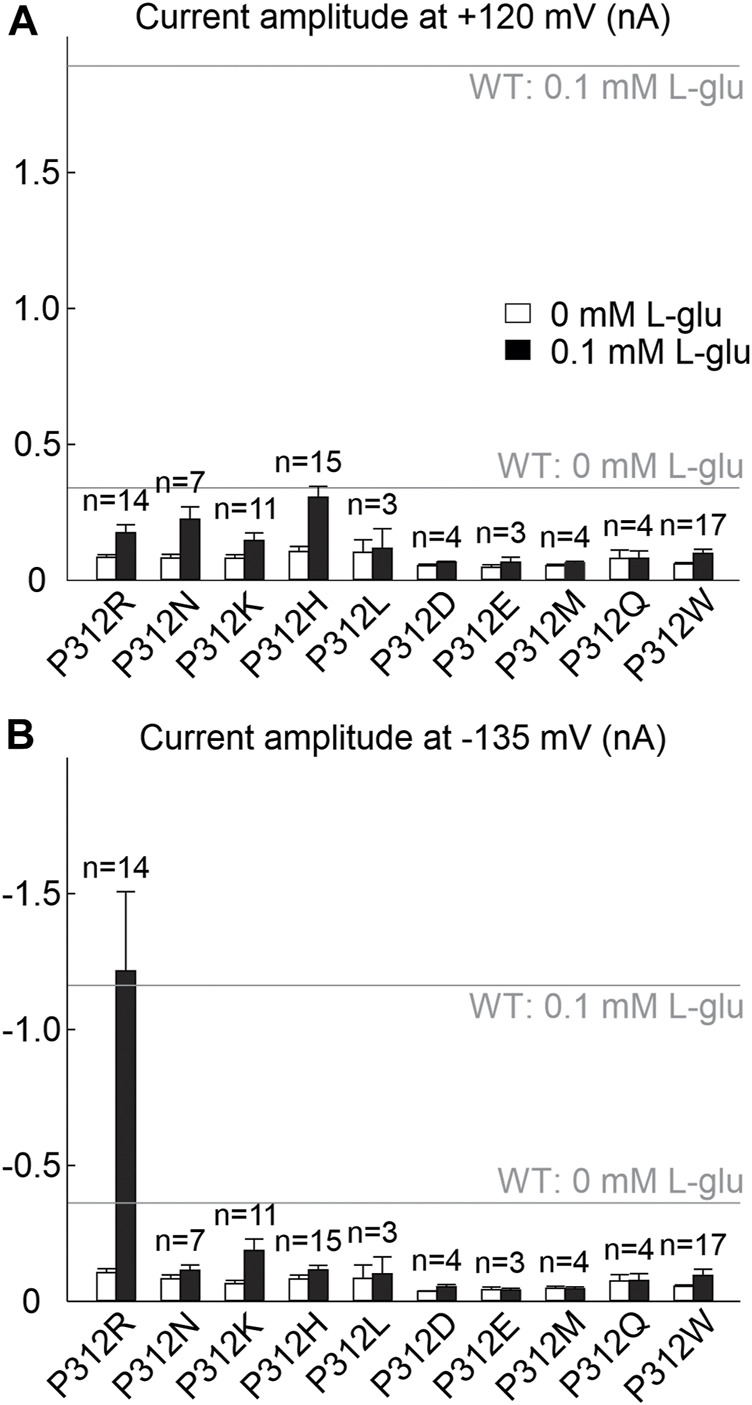
Many amino acid substitutions of P312 reduce EAAT4 anion current amplitudes. **(A, B)** Current amplitudes at +120 mV **(A)** or −135 mV **(B)** from HEK293T cells expressing WT or mutant EAAT4 with KNO_3_-based internal solutions and NaNO_3_-based external solutions after application of 0.1 mM L-glutamate (lower row). Dotted lines indicate background currents measured in untransfected cells with external glutamate.

We substituted proline 312 in EAAT4 (Figure 1A) by alanine, arginine, asparagine, aspartate, cysteine, glutamate, glutamine, glycine, histidine, leucine, lysine, methionine, serine or tryptophan and expressed WT and mutant EAAT4 as mYFP fusion protein in HEK293T cells. EAATs transport three Na^+^, one glutamate and one H^+^ in exchange with one K^+^ (Zerangue and Kavanaugh, 1996), and we therefore studied all mutants in experiments with cells dialyzed with K^+^-containing solutions and externally perfused with Na^+^-containing solutions with or without L-glutamate. In the following, these ionic conditions are referred to as uptake condition. NO_3_^−^-based internal and external solutions were used to increase anion currents (Wadiche and Kavanaugh, 1998; Melzer et al., 2003). Only expression of P312H, P312K, P312N and P312R EAAT4 resulted in measurable L-glutamate-dependent anion currents under these conditions (Figures 1, 2). In cells expressing P312H and P312K EAAT4, current amplitudes were very small (Supplementary Figure S1), and we therefore restricted a detailed functional analysis to P312R and P312N EAAT4.

Figure 1C shows representative current recordings from cells expressing WT, P312R or P312N EAAT4 before (upper row) or after (lower row) L-glutamate application. Currents were small in the absence of L-glutamate, and significantly larger than background currents (given as solid lines in Figure 1D) only for WT or P312R EAAT4. For WT as well as for mutant EAAT4, anion currents were time-independent in the absence of L-glutamate, with larger conductances in the negative voltage ranges than in the positive voltage ranges. Application of L-glutamate increased current amplitudes, with distinct time and voltage dependences for WT and mutant EAAT4 in the presence of L-glutamate. WT and P312N EAAT4 anion currents showed bidirectional rectification, with macroscopic conductances increasing upon hyper- or depolarization (Figure 1D). WT EAAT4 exhibits a characteristic deactivation upon hyperpolarizing voltage steps (Kovermann et al., 2010). P312R EAAT4 anion currents increase in time and voltage-dependent manner at negative voltages. Under these ionic conditions, current amplitudes were comparable for WT and P312R EAAT4, but tenfold smaller in P312N EAAT4. Supplementary Figure S2 shows L-glutamate dependences for WT and mutant EAAT4 anion currents measured at symmetrical NO_3_^−^. Dose-response curves were fit with Michaelis-Menten relationships with K_M_ constants in the low µM range (WT: K_M_ = 2.9 ± 1.2 μM at −135 mV, K_M_ = 9.5 ± 2.6 μM at 120 mV/P312R: K_M_ = 0.03 ± 0.02 μM at −135 mV, K_M_ = 0.4 ± 0.2 μM at 120 mV/P312N: K_M_ = 0.03 ± 0.01 μM at −135 mV, K_M_ = 1.5 ± 1.0 μM at 120 mV) both strongly decrease L-glutamate K_M_s. Increased L-glutamate affinity was already described for P290R EAAT1 (Winter et al., 2012).

### P312R and P312N impair EAAT4 glutamate transporter biogenesis and trafficking

**FIGURE 3:**
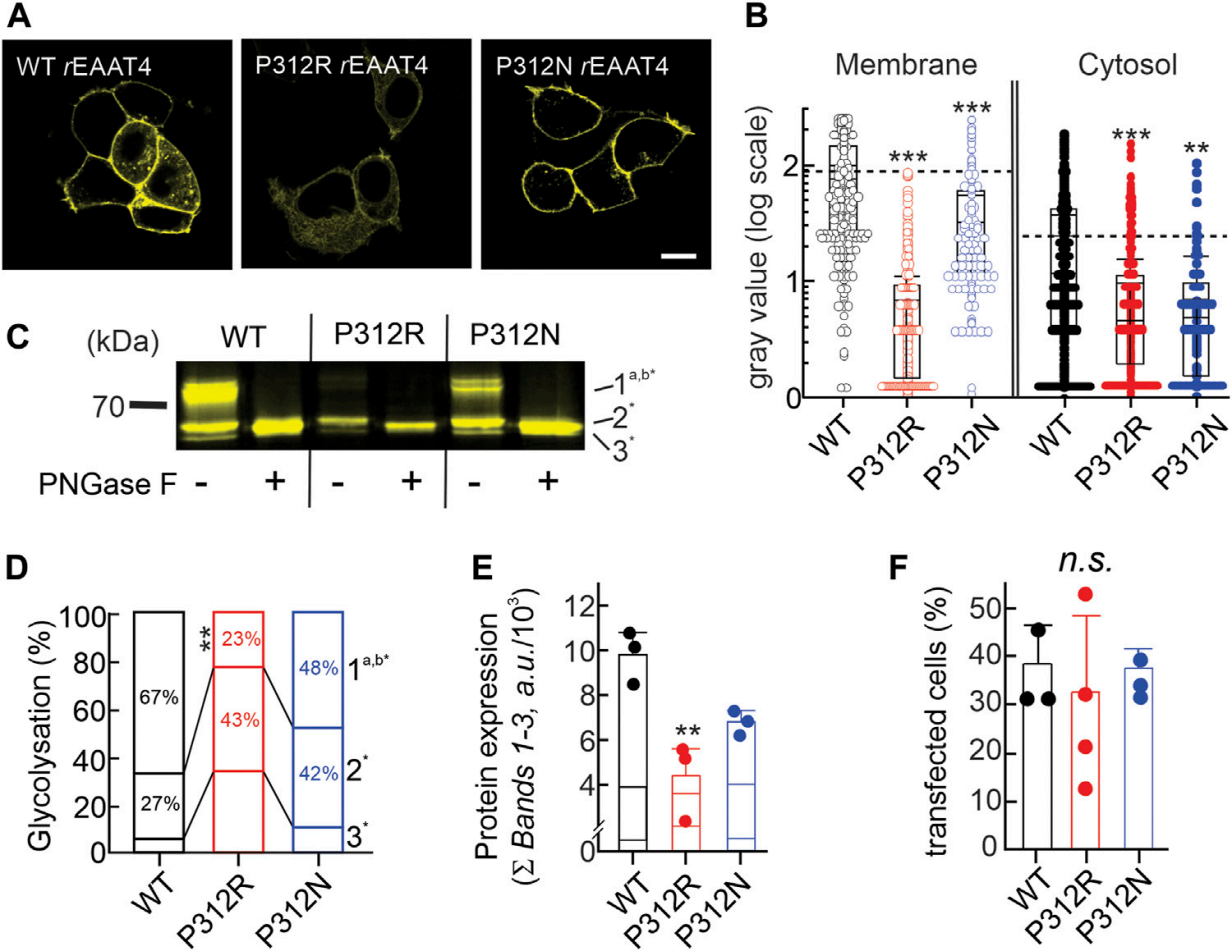
Mutating proline 312 reduces EAAT4 protein expression and modifies intracellular trafficking. **(A)** Representative confocal images of HEK293T cells expressing WT or mutant EAAT4-mYFP fusion proteins. **(B)** Box-plot analysis of fluorescence intensities from membranes and cytosolic areas. **(C)** Representative fluorescence scan of a SDS-PAGE loaded with equal amounts of whole cell lysates expressing WT or mutant EAAT4, either pretreated with PNGase F (+) or not (−). **(D)** Stacked bar graph showing the distribution of glycolysation states for WT or mutant EAAT4. **(E)** Mean protein expression levels determined from sums of fluorescence band intensities of all glycolysated fractions (Ʃ bands 1–3). **(F)** Transfection efficiencies given as percentages of transfected cells for three to four transfections per tested variant.

**FIGURE 4:**
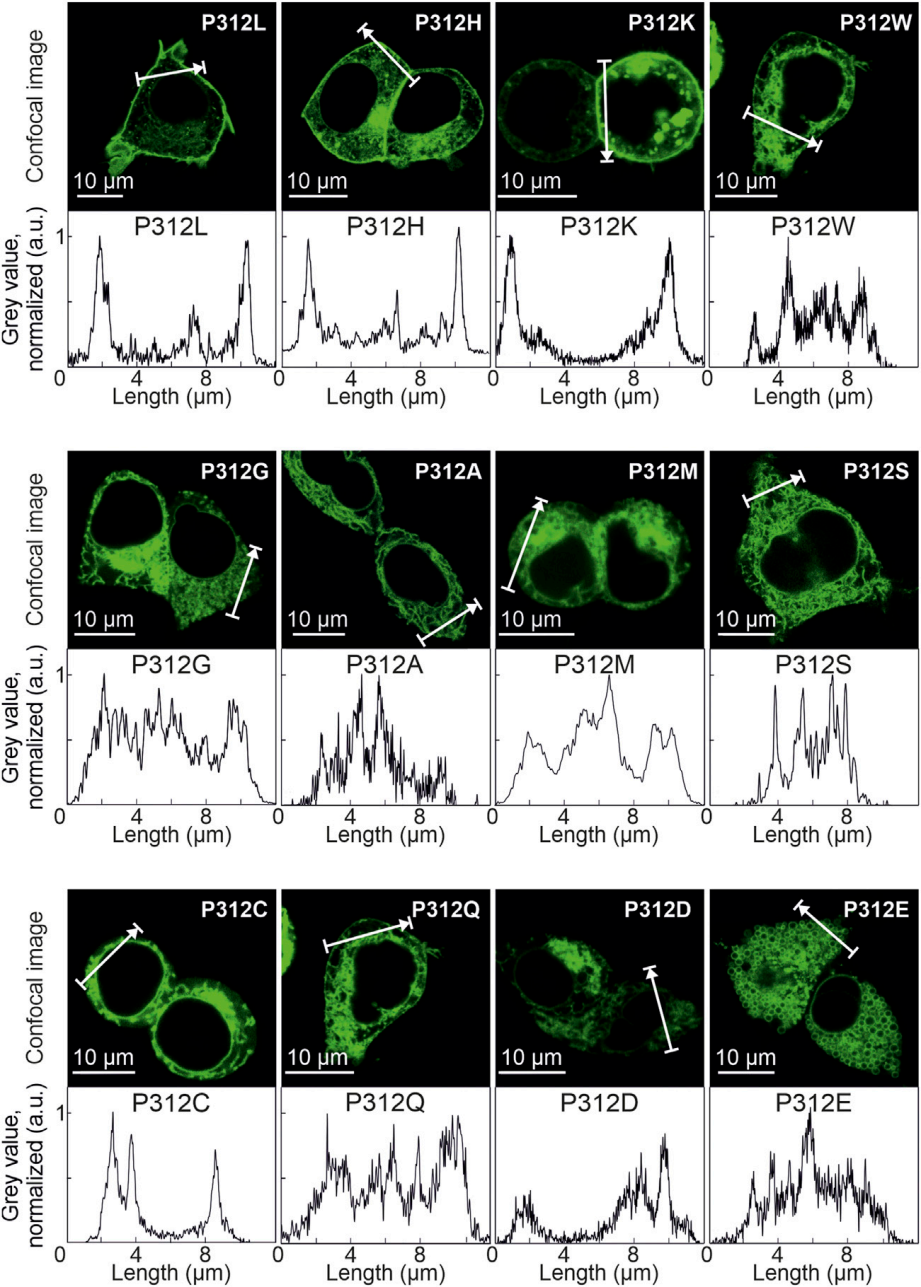
Various amino acid substitutions of P312 cause intracellular retention of EAAT4. Confocal images and corresponding intensity profiles of HEK293T cells expressing mutant mYFP-EAAT4 fusion proteins.

Figure 3A depicts representative confocal images of cells expressing WT, P312R or P312N mYFP-EAAT4. We observed predominant surface membrane insertion for WT as well as for these mutant EAAT4, however, with large differences in total fluorescence intensities. The majority of the other mutants were retained in intracellular compartments (Figure 4). Separating fluorescences in the surface membrane from cytosolic mYFP revealed approximately tenfold lower fluorescence levels for P312R in or close to the surface membrane and twofold lower levels for P312N than for WT EAAT4 (Figure 3B). There are also lower amounts of mutant transporters in the cytosol (Figure 3B).

Resolving WT and mutant mYFP-EAAT4 fusion proteins after expression in HEK293T cells by reducing SDS-PAGE (Figure 3C) revealed triple fluorescence bands with molecular sizes of approximately 60–70 kDa. Deglycosylation with PNGase F removes the upper and the middle molecular weight bands, assigning non-, core- and complex glycosylated states to the three bands (Figure 3C). P312N and P312R both reduces complex glycosylation of EAAT4, with more pronounced effects by P312R than by P312N (Figure 3D). Integrating fluorescence amplitudes of all three bands provides averaged cellular expression levels and revealed reduced expression levels of P312R and P312N mYFP-EAAT4 (Figure 3E). In such biochemical experiments, we did not distinguish transfected from non-transfected cells, and the observed differences in protein amounts may thus be either caused by reduced cellular protein expression or by reduced percentages of transfected cells. However, no differences in transfection efficiencies were observed between transfections with WT or mutant EAAT4 (Figure 3F). We conclude that P312R and P312N reduce expression levels and surface membrane insertion of EAAT4.

### P312R endows EAAT4 with a unique K^+^ dependence

Our group has studied multiple EAATs using K^+^-free and Na^+^-based intracellular solutions (Melzer et al., 2003; Kovermann et al., 2010; Schneider et al., 2014). Under these conditions, obligate K^+^-dependent retranslocation and L-glutamate transport are not possible (Kortzak et al., 2019), and EAATs are expected to accumulate in an inward-facing conformation that is inaccessible to external L-glutamate. However, we observed L-glutamate-sensitive anion currents for all tested EAATs with Na^+^-based intracellular solutions (Melzer et al., 2003; Leinenweber et al., 2011; Schneider et al., 2014), possibly due to re-translocation after rebinding of L-glutamate from the internal side. Alternatively, there might exist slow Na^+^-bound only translocation processes (Bergles et al., 2002). Since Na^+^ binding in the absence of L-glutamate stabilizes HP2 in an open state (Alleva et al., 2020; Alleva et al., 2021), it is currently unclear how such translocations occur. Although we do not fully understand the mechanisms of anion channel activation in cells internally dialyzed with Na^+^-based solutions, comparison of WT and mutant EAAT4 revealed unique properties of P312R EAAT4.

**FIGURE 5:**
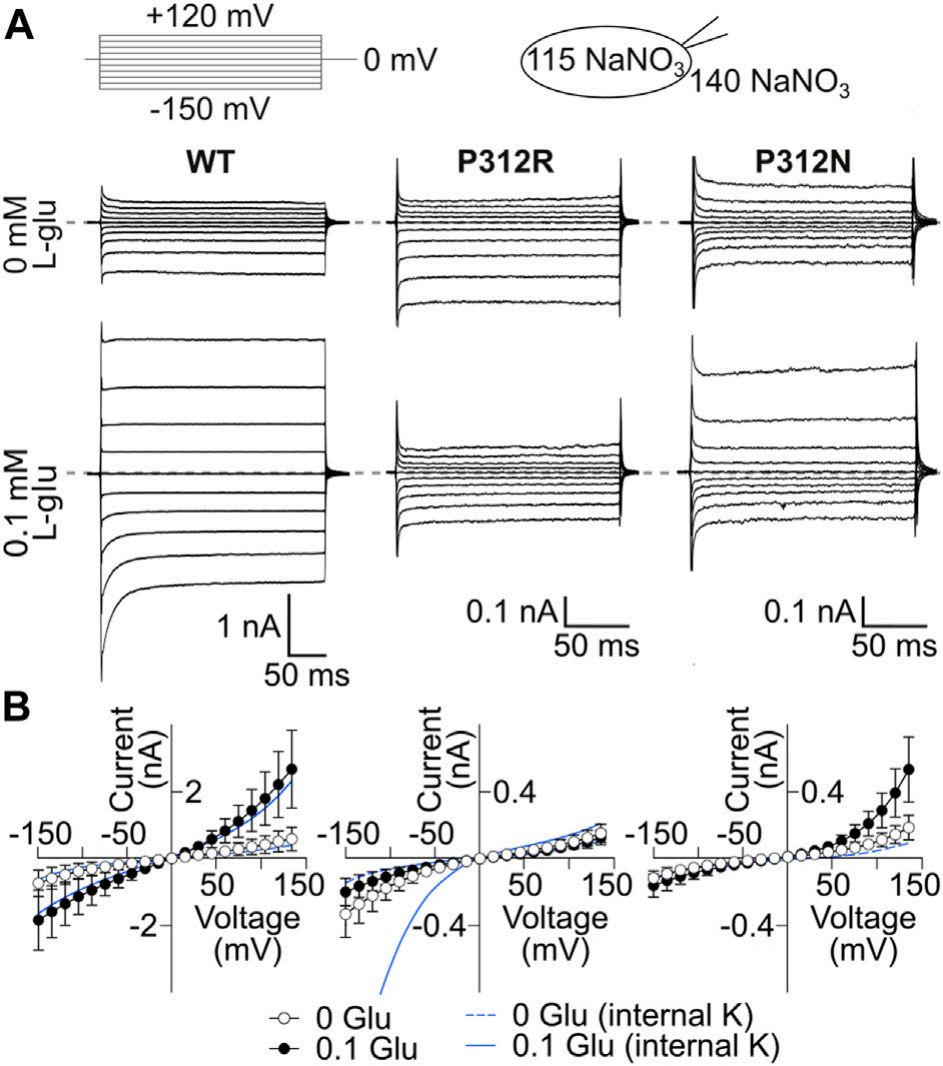
P312R makes EAAT4 anion currents glutamate-insensitive in the presence of internal Na^+^. **(A)** Representative current recordings from HEK293T cells expressing WT (left), P312R (middle) or P312N (right) *r*EAAT4 with a NaNO_3_-based internal solution and a NaNO_3_-based external solution before (upper row) and after application of 0.1 mM L-glutamate (lower row). **(B)** Voltage dependence of mean anion current amplitudes from cells expressing EAAT4 variants obtained in cells intracellularly dialyzed with NaNO_3_-based solutions. Data are given as the means ± *C.I*.; *n* ≥ 7. Background currents obtained with untransfected HEK293T cells are shown as solid line in the presence of glutamate and dashed line in the absence of it. Blue lines provide current-voltage relationships for cells expressing WT and mutant EAAT4 and dialyzed with a K^+^-based internal solution.

WT and mutant EAAT4 anion currents exhibit different substrate, time and voltage dependences when tested with Na^+^ as major internal cation as compared to uptake conditions (Figure 5A). These changes reflect the reduced number of states the transporters can occupy in the absence of internal K^+^. Anion current amplitudes were comparable in cells expressing WT or P312N EAAT4 for K^+^-and Na^+^-based internal solutions, but were substantially smaller under internal Na^+^ conditions (Figure 5B) than under uptake conditions for P312R EAAT4 (Figure 1D). This result suggests that anion conduction within the K^+^ hemicycle is especially important for gain-of-anion channel function of this mutant. Moreover, whereas anion currents were stimulated by L-glutamate for WT or P312N EAAT4 with both tested internal solutions, we observed no L-glutamate-induced enhancement of P312R EAAT4 currents for cells dialyzed with Na^+^-based solutions (Figure 5B).

### P312N and P312R impair EAAT4 glutamate transport

**FIGURE 6:**
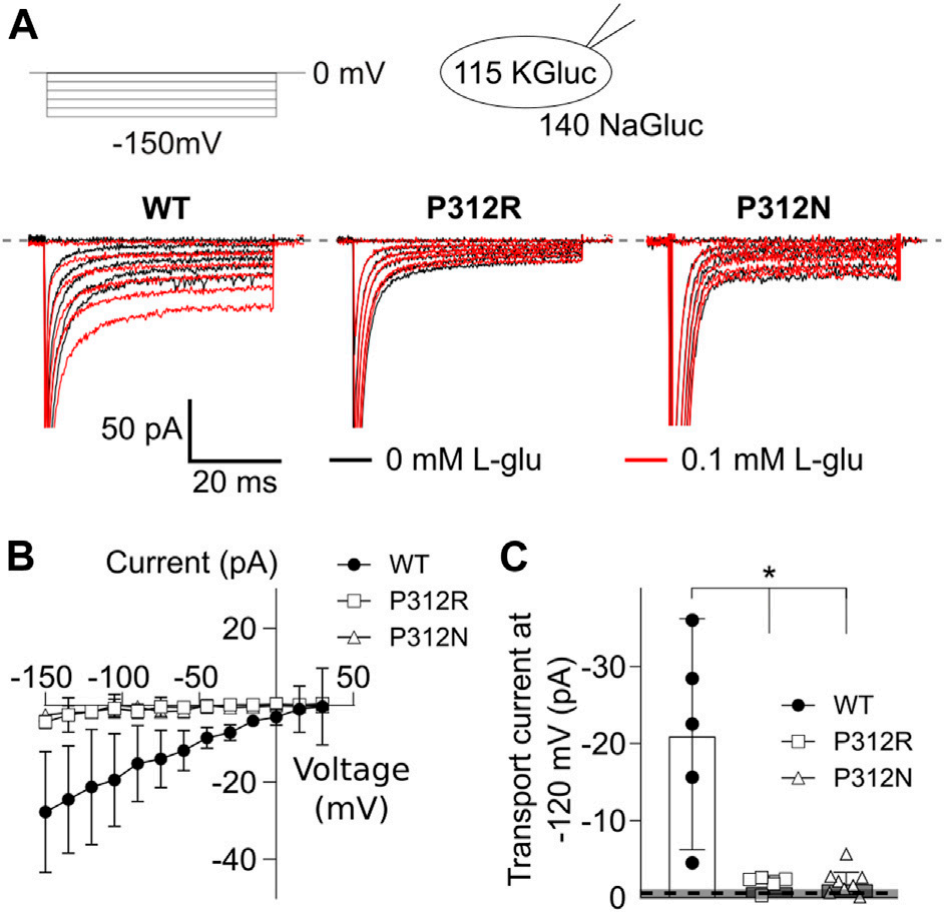
Mutations in P312 modify glutamate transport by EAAT4. **(A)** Representative current recordings from HEK293T cells expressing WT (left), P312R (middle) or P312N (right) EAAT4 with K-D-gluconate-based internal solution and Na-D-gluconate-based external solution before (black traces) and after application of 0.1 mM L-glutamate (red traces). **(B)** Voltage dependence of mean L-glutamate transport current amplitudes from cells expressing WT, P312R, or P312N EAAT4. Data are given as the means ± *C.I*.; *n* ≥ 4. **(C)** Comparison of WT, P312R and P312N EAAT4 transport currents at −120 mV. Control data from untransfected cells (means ± CI) are given as grey box.

EAATs mediates the stoichiometrically coupled transport of one glutamate, three Na^+^ and one H^+^, in exchange with one K^+^. One transport cycle is therefore associated with the transport of two positive elementary charges across the membrane. Glutamate transport therefore generates a current, the so-called transport current, which can be quantified as L-glutamate-sensitive current component in internal and external solutions, in which permeant anions were completely substituted with gluconate (Figure 6A). EAAT4 is a prototypical low capacity glutamate transporter (Fairman et al., 1995; Mim et al., 2005), and EAAT4 L-glutamate transport currents are usually very small (Figure 6B). Transport currents measured approximately 30.4 ± 5.7 pA (*n* = 6, WT), 4.4 ± 0.6 pA (*n* = 5, P312R), and 1.8 ± 0.1 pA (*n* = 5, P312N) at −120 mV (Figure 6C). We conclude that P312R and P312N virtually abolish transport of L-glutamate.

### Fast substrate application reveals changes in the glutamate transport cycle in mutant EAAT4

The effects of P312 R/N on EAAT4 anion currents under uptake and under internal Na^+^ conditions as well as impaired L-glutamate uptake by mutant transporters indicate that amino acid substitutions at position 312 result in major modifications of the EAAT4 transport cycle (Figure 6A). To quantitatively describe these changes, we analyzed WT and mutant EAAT4 anion current responses to fast piezo-driven substrate applications (Franke et al., 1987; Jonas and Sakmann, 1992; Otis and Kavanaugh, 2000) for two internal solutions. Whereas L-glutamate application with internal K^+^ will report on all possible anion-conducting conditions, intracellular dialysis with Na^+^ + L-glutamate will restrict transitions to Na^+^/L-glutamate bound translocation. Similarly, K^+^-application to cells dialyzed with K^+^-based internal solutions will provide insights into K^+^-bound re-translocation as well as K^+^-bound or *apo* state inward- or outward-facing conformations.

**FIGURE 7:**
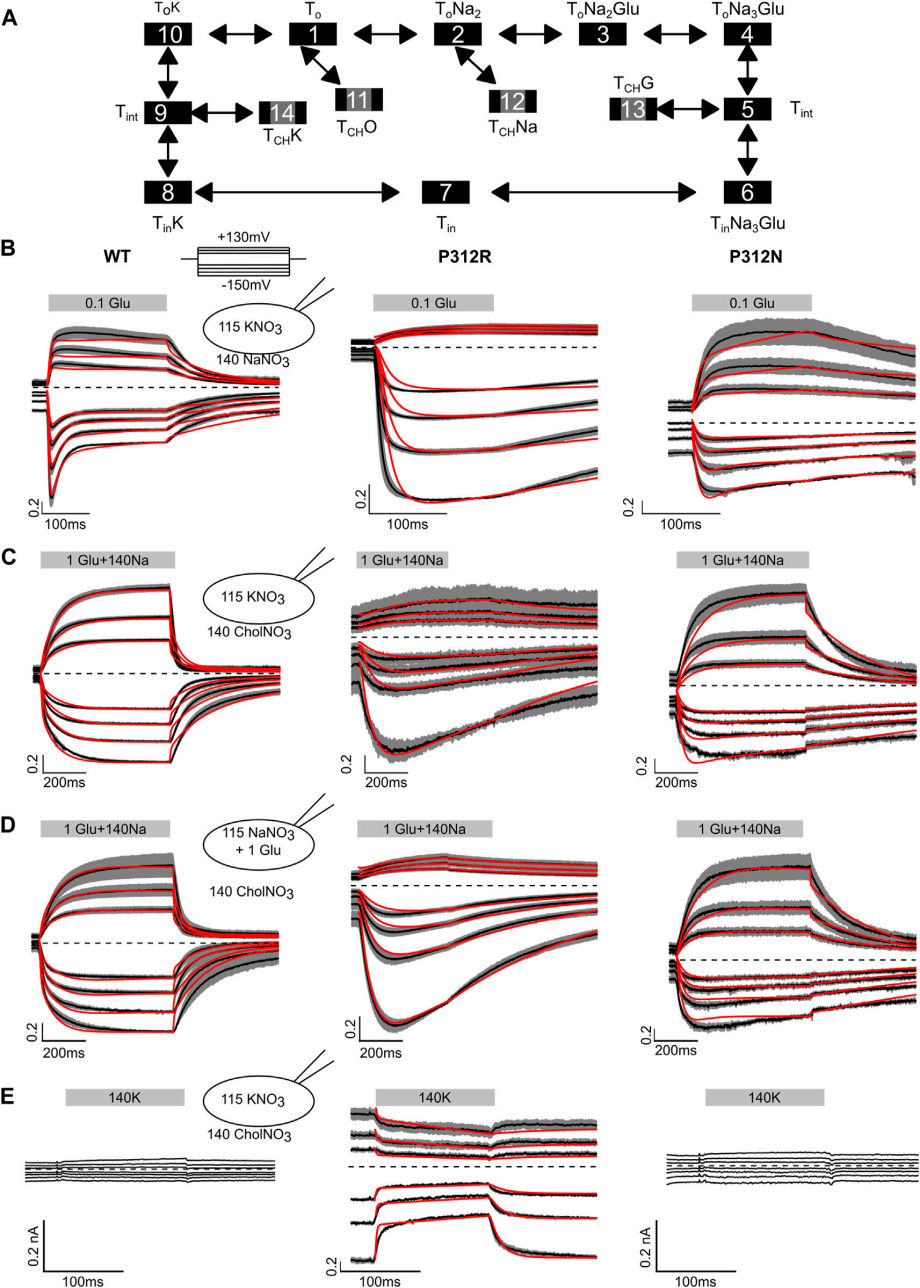
Fast application experiments with WT, P312R or P312N EAAT4. **(A)** Kinetic scheme for EAAT glutamate transport. **(B–D)** Normalized mean (black lines) of all current recordings from HEK293T cells expressing WT (left), P312R (middle) or P312N (right) EAAT4 dialyzed either with KNO_3_-based **(B, C, E)** or NaNO_3_ + L-glutamate-based **(D)** internal solution upon rapid application of L-glutamate **(B)**, Na^+^ + L-glutamate **(C, D)** or K^+^ **(E)**. Shaded area indicate confidence intervals, and red lines provide the time-course of simulated normalized open probabilities. Absolute WT and P312N EAAT4 currents **(E)** are indistinguishable from background; we therefore did not fit such currents.

Figure 7 shows averaged WT, P312R and P312N EAAT4 current responses (±*C.I.*, *n* = 10) to fast solution exchanges. Cells were held at 0 mV, and voltage steps between −150 mV and +150 mV were applied. After reaching steady-state current amplitudes under the original ionic condition the external solution was quickly changed using a two-barreled application pipette attached to a piezoelectric bimorph (Carbone and Plested, 2012) and subsequently changed back to the original solution. The duration of solution application is given by a grey box containing the content of the perfused solution. In Figure 7B, 0.1 mM L-glutamate was applied to cells continuously perfused with a Na^+^-based solution and internally dialyzed with K^+^-based solutions. WT EAAT4 anion currents respond with fast increase followed by slight decrease in the positive range, and with biphasic responses consisting of fast increases and slower decreases at negative voltages, resembling earlier experiments using glutamate uncaging (Mim et al., 2005). For P312R EAAT4, current responses were inwardly rectifying; with fast activation and without deactivation at negative voltages. For P312N EAAT4, L-glutamate application results in activation to large current amplitudes at positive potentials and smaller changes in current amplitudes at negative voltages. Relative L-glutamate-induced changes in current amplitudes were larger for WT and P312R than for P312N EAAT4 currents. After removal of L-glutamate, transporters return via K^+^-bound retranslocation into outward-facing conformations. For both mutant transporters, L-glutamate removal results in much slower decays of current amplitudes than for WT transporters.

Fast application of Na^+^ together with L-glutamate to WT EAAT4 transporters equilibrated in choline-based solutions and dialyzed with K^+^-based solutions elicits slow activation at positive as well as at negative voltages (Figure 7C), likely reflecting slow conformational changes associated with Na^+^ binding to *apo* state glutamate transporters (Ewers et al., 2013; Alleva et al., 2020). Changing to a Na^+^-based solution with saturating L-glutamate concentrations from a choline-based external solution resulted in comparable time courses under uptake conditions, i.e., in cells internally dialyzed with K^+^ (Figure 7B), and under exchange conditions, i.e., in cells dialyzed with Na^+^ + L-glutamate (Figure 7D). For P312R EAAT4, current responses were inwardly rectifying upon application of L-glutamate alone (Figure 7B) or upon application of Na^+^ + L-glutamate application, under transport (Figure 7C) as well as under exchange conditions (Figure 7C). For both internal solutions, cells expressing P312R EAAT4 display currents exceeding 400 pA at external choline^+^. Under these conditions, transporters are expected to be in the outward facing conformations. Current amplitudes clearly above background indicate anion conducting states in this conformation. Currents are comparable in external Na^+^ and in external choline^+^; with K^+^-based internal solutions as well as with internal Na^+^/L-glutamate. P312N EAAT4 anion currents are outwardly rectifying also for these conditions, and Na^+^ application resulted in slow activation. There are substantially smaller currents with choline than with Na^+^ as main external cation, indicating major contributions of P312N EAAT4 anion conducting states that can be accessed from Na^+^-bound, but not from *apo* outward facing conformations.

Figure 7E depicts current responses of cells intracellularly dialyzed with K^+^-based solutions to changes from K^+^-free to K^+^-containing solutions. Neither in cells expressing WT nor P312N EAAT4 any currents were observed under these conditions. In contrast, there exist substantial P312R EAAT4 anion currents in K^+^-free solutions that were reduced upon application of external K^+^. These results indicate that P312R induce a hitherto unknown open anion channel conformation that is accessible from *apo* outward facing conformation.

Kinetic modeling reveals distinct changes in substrate association and translocation as well as in anion channel opening in mutant EAAT4. To test which conformational changes are affected by the P312 mutants, we built a kinetic model (Figure 7A) and fitted simulated currents to current responses to fast substrate application (Figures 7B–E; Table 1). The model is based on a published EAAT2/Glt-1 model (Bergles et al., 2002; Machtens et al., 2011; Kovermann et al., 2017). Since we did not study effects of varying internal or external pHs, we lumped H^+^-free and H^+^-bound states together. Moreover, we only distinguished inward facing conformations bound to Na^+^ and L-glutamate, bound to K^+^ or in the *apo* state. Open anion conducting conformations (numbers marked in grey) were assumed to be accessible from intermediate conformations with bound Na^+^ and L-glutamate or bound K^+^, in agreement with recent MD results demonstrating that anion channels with functional properties that resemble experimental results open during lateral movements of the transport domain from intermediate positions (Machtens et al., 2015; Cheng et al., 2017). There is experimental evidence that supports branching anion channel states: rapid glutamate application results in a faster onset of EAAT3 glutamate transport currents than of anion currents (Grewer et al., 2000). Since this result is in disagreement with EAAT anion conducting states within translocation transition that were recently suggested (Chen et al., 2021), we decided not to include such states.

**TABLE 1:** Rate constants of the transport process and of channel gating at 0 mV. Electrogenic reactions are defined by z values, which correspond to the fraction of the electric field the charge is moved across the membrane.

Transition	WT			P312R			P312N		
	Forward	Backward	z	Forward	Backward	z	Forward	Backward	z
**T**_**o**_ **-T**_**o**_ **Na2**	**80 M** ^**-1**^ **s** ^**-1**^	**2 s** ^**-1**^	0.1	**80 M** ^**-1**^ **s** ^**-1**^	**2 s** ^**-1**^	0.2	**80 M** ^**-1**^ **s** ^**-1**^	12.4** **s^-1^	0.08
**T**_**o**_ **Na2-T**_**o**_ **Na2Glu**	**1.0•10** ^**6**^ ** M** ^**-1**^ **s** ^**-1**^	9,800** **s^-1^	0	**1.0•10** ^**6**^ ** M** ^**-1**^ **s** ^**-1**^	1400** **s^-1^	0	**1.0•10** ^**6**^ ** M** ^**-1**^ **s** ^**-1**^	8.8** **s^-1^	0
**T**_**o**_ **Na2Glu-T**_**o**_ **Na3Glu**	**2.5 •10** ^**4**^ ** M** ^**-1**^ **s** ^**-1**^	**1000 s** ^**-1**^	0.0	**2.5 •10** ^**4**^ ** M** ^**-1**^ **s** ^**-1**^	0.9** **s^-1^	0.13	277 M^-1^s^-1^	**1000 s** ^**-1**^	0.0
**T**_**o**_ **Na3Glu-T**_**int**_	781** **s^-1^	**200 s** ^**-1**^	0.2	0.6** **s^-1^	**200 s** ^**-1**^	0.2	4,625** **s^-1^	3053** **s^-1^	0.11
**T**_**int**_ **-T**_**in**_ **Na3Glu**	**200 s** ^**-1**^	33** **s^-1^	0.07	**200 s** ^**-1**^	16.8** **s^-1^	0.31	5,627** **s^-1^	15** **s^-1^	0.14
**T**_**in**_ **Na3Glu-T**_**in**_	**0.3 s** ^**-1**^	9.7 •10^6^ M^-3^ s^-1^	0.7	11.7** **s^-1^	780.1** **M^-3^s^-1^	0.5	**0.3 s** ^**-1**^	9.8•10^5^ M^-3^s^-1^	0.78
**T**_**in**_ **-T**_**in**_ **K**	**10,000 M** ^**-1**^ **s** ^**-1**^	**1000 s** ^**-1**^	0	618 M^-1^s^-1^	2,130** **s^-1^	0	**10,000 M** ^**-1**^ **s** ^**-1**^	**1000 s** ^**-1**^	0
**T**_**in**_ **K-T**_**int**_	**1 s** ^**-1**^	0.004** **s^-1^	0.76	0.5** **s^-1^	4** **s^-1^	0.2	**1 s** ^**-1**^	0.123** **s^-1^	0.73
**T**_**int**_ **-T**_**o**_ **K**	**50 s** ^**-1**^	**1000 s** ^**-1**^	0.13	55** **s^-1^	**1000 s** ^**-1**^	0.43	**50 s** ^**-1**^	**1000 s** ^**-1**^	0.13
**T**_**o**_ **K-T**_**o**_	**13000 s** ^**-1**^	**1.2•10** ^**6**^ ** M^-1^ s^-1^**	0	594** **s^-1^	9.9 •10^6^ M^-1^s^-1^	0	**13000 s** ^**-1**^	**1,200,000 M** ^**-1**^ **s** ^**-1**^	0
**T**_**int**_ **Glu-T**_**CH**_ **F**	0.77** s** ^**-1**^	775** **s^-1^	0	925** **s^-1^	12,081** **s^-1^	0	1737** **s^-1^	19807	0
**T**_**int**_ **K-T**_**CH**_ **K**	0	0	0	0.4** **s^-1^	5.5** **s^-1^	0	0	0	0
**T**_**o**_ **-T**_**CH**_ **O**	0	0	0	0.1** **s^-1^	3591** **s^-1^	0	0	0	0
**T**_**o**_ **Na2-T**_**CH**_ **Na**	0.00009** s** ^**-1**^	6.0866** **s-1	0	8.6 •10^–8^ ** **s^-1^	2,102** **s^-1^	0	0.4** **s^-1^	7,230** **s^-1^	0

Bold values were constrained during optimization.

To account for EAAT anion conductance with external Na^+^ in absence of L-glutamate an open channel state was linked to the outward-facing state with two associated Na^+^ ions. To explain the inhibition of current in the K^+^ application experiments of P312R, an additional channel opening from the outward-facing *apo* state was inserted. Voltage dependences were optimized by fitting current responses at different voltages. Unitary currents were assumed to changes linearly with voltage (Kovermann et al., 2010). P290R is not modifying the unitary current amplitudes of EAAT1 (Winter et al., 2012), and P290 is not contributing to forming the anion conduction pathway (Machtens et al., 2015). We therefore assumed that P312R and P312N EAAT4 anion channels also exhibit voltage-independent unitary conductances and that distinct rectification of WT and mutant EAAT4 is due to separate voltage dependent open probabilities.

**FIGURE 8:**
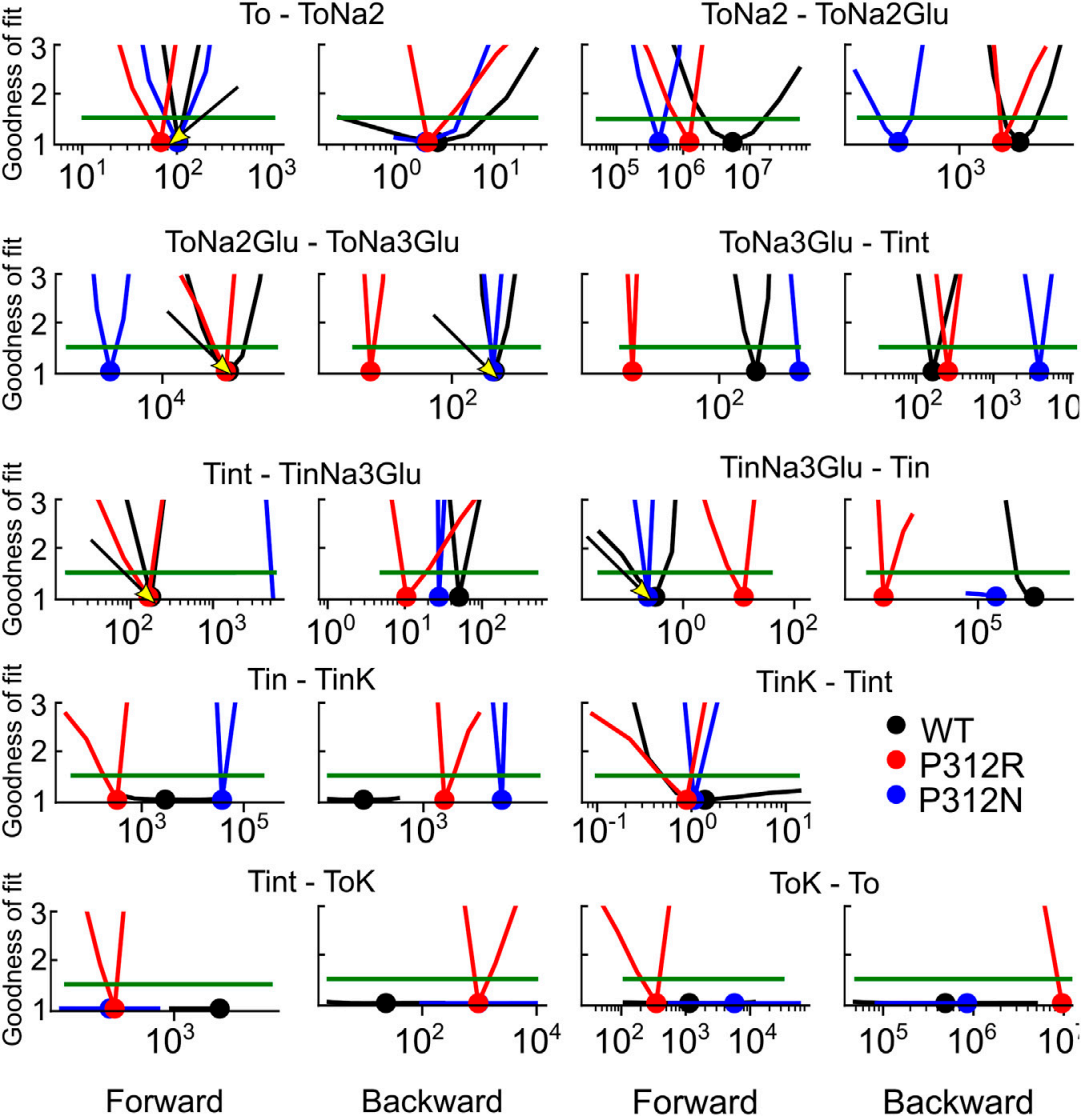
Iterative parameter optimization. Changes of the goodness of fit upon variation of individual parameters, while remaining rates were kept constant after the initial fit with no constraints. The goodness of fit was determined for fits on WT and mutant EAAT4 current traces and normalized to the minimum value. Dots indicate the position of the minimum, and green lines indicate 50% increase in the relative goodness of fit. Arrows indicate values, to which rates were constrained before starting the 2nd fitting iteration with reduced number of free parameters. This analysis was not performed for T_int_—T_int_K, because this rate is restrained by detailed balance.

We used an iterative procedure to optimize fit parameters. Initially, experimental data were fitted for each of the three constructs. Subsequently, the thus obtained parameters were individually varied, with all other parameters kept constant, and changes in the goodness of fit were calculated as function of the parameter variation (Figure 8). This analysis permits identification of the parameter interval, in which the goodness of fit is increased by less than 50% of the minimum value (the fit cutoffs are given by green lines in Figure 8). For some parameters, these intervals overlapped for two or three constructs. In such cases, we concluded that fit parameters were the same and fixed them to the mean of both values (given as arrow in Figure 8) in subsequent iterations. If forward and backward rate of one transition were identified by this criterion, we fixed only one of them. In the next fit iteration, the remaining free parameters were optimized in new fitting procedures, followed again by variation of individual parameters. These procedures were repeated three times. Parameter, which were fixed during this procedure, are provided in bold in Table 1.

**FIGURE 9:**
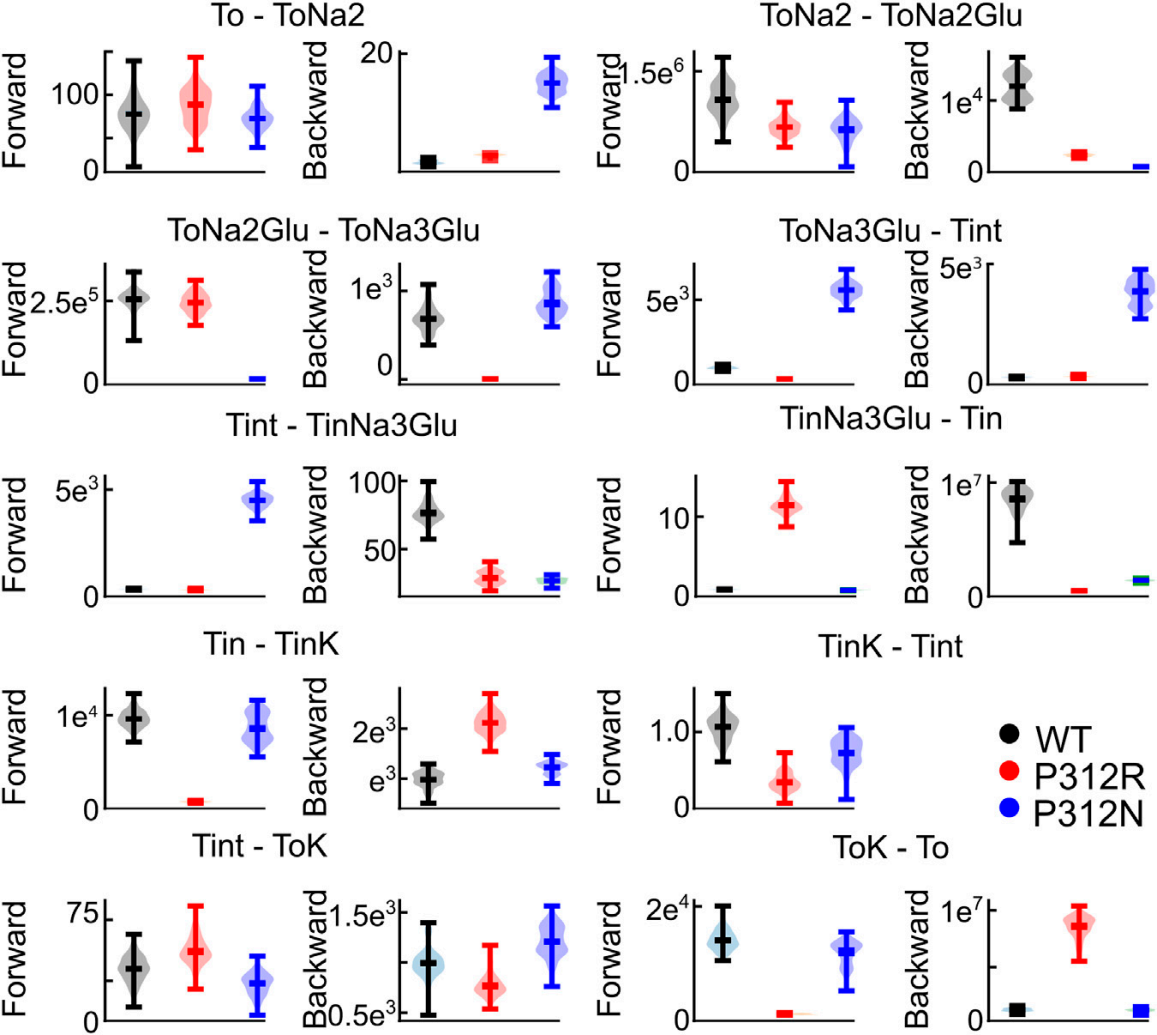
Not all fit parameter are equally well defined. Violin plots of parameters with a goodness of fit that differs by less than 25% from the optimum fit. This analysis was not performed for T_int_ - T_int_K, because this rate is restrained by detailed balance.

We tested the accuracy of our fitting results in an additional procedure, in which all fit parameters were randomly modified. Fit parameters that decreased the goodness of fit by less than 25% were collected, providing a distribution of fit parameters that permit fitting experimental data with similar accuracy (Figure 9). This analysis demonstrated that rates of certain transitions, e.g., glutamate binding or K^+^-bound translocation, are not very well defined, with a large range of parameters equally well describing the data. On the other hand, this analysis also identifies parameters, with mutant values that are far away from WT values and have a big impact on the fit quality. There are major alterations in both hemicycles by P312R (Table 1). P312R reduces the unbinding rate of glutamate and the third Na^+^ as well as translocation rates from T_o_Na_3_Glu to intermediate and to inward facing states within the Na^+^/L-glutamate hemicycle. Moreover, P312R impairs association of substrates to T_i_Na_3_GH and dissociation of K^+^ from the outward-facing conformation increases K^+^ binding to inward-facing conformations. P312N reduces Na^+^ binding to *apo* as well as to T_o_Na_2_GH and L-glutamate unbinding from the outward-facing conformation. It speeds up glutamate translocation rates, but leaves translocation rates of the K+ hemicycle unaffected (Table 1; Figure 9). Whereas impaired L-glutamate bound translocation as well as K^+^-bound retranslocation account for reduced L-glutamate transport by P312R EAAT4, slowed substrate release to the cytoplasm reduces P312N EAAT4 glutamate transport.

**FIGURE 10:**
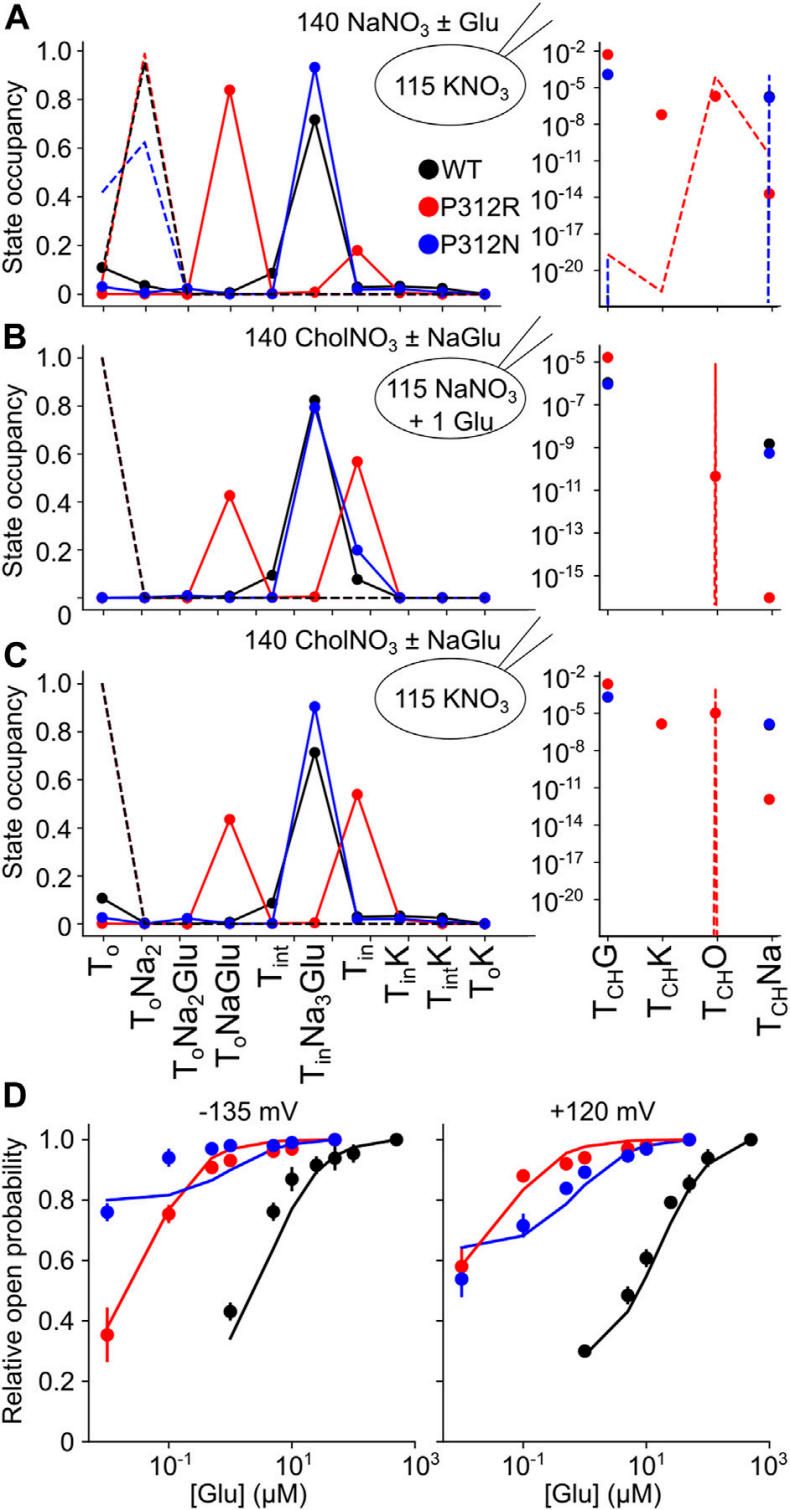
Gain-of-anion channel function of P312R EAAT4 is exclusively due to *apo* state channel opening. **(A–C)** Simulated residence probabilities for WT, P312R or P312N EAAT4 in states of the transport cycle (left) or in open anion channel (right) conformations for indicated ion and substrate conditions before (dashed lines) or after (symbols) solution exchange. **(D)** Experimental and simulated glutamate titration curves.

The thus optimized kinetic model correctly reproduces experimentally observed anion currents for all tested constructs (Figures 7B–E) and accounts for experimentally observed changes in L-glutamate transport (WT 2.5/s, P312N 0.05/s, P312R 10^–6^/s). Figure 10 depicts predicted residence probabilities for transport (left panels) or anion conducting states (right panels) before and after substrate application for all internal conditions shown in Figure 5. In the presence of external glutamate, WT and P312N EAAT4 reside predominantly in the fully bound inward facing conformation. This distribution is shifted towards the outward facing fully bound and the inward-facing *apo* states in P312R EAAT4 (Figures 10A–C). Increased anion currents by P312R EAAT4 are caused by additional occupation of anion conducting states in the *apo* state; the probability of assuming the Na^+^-bound only anion conducting states is decreased in P312R EAAT4 (Figures 9A–C). There are only slight modifications of anion channel occupations by P312N.

**FIGURE 11:**
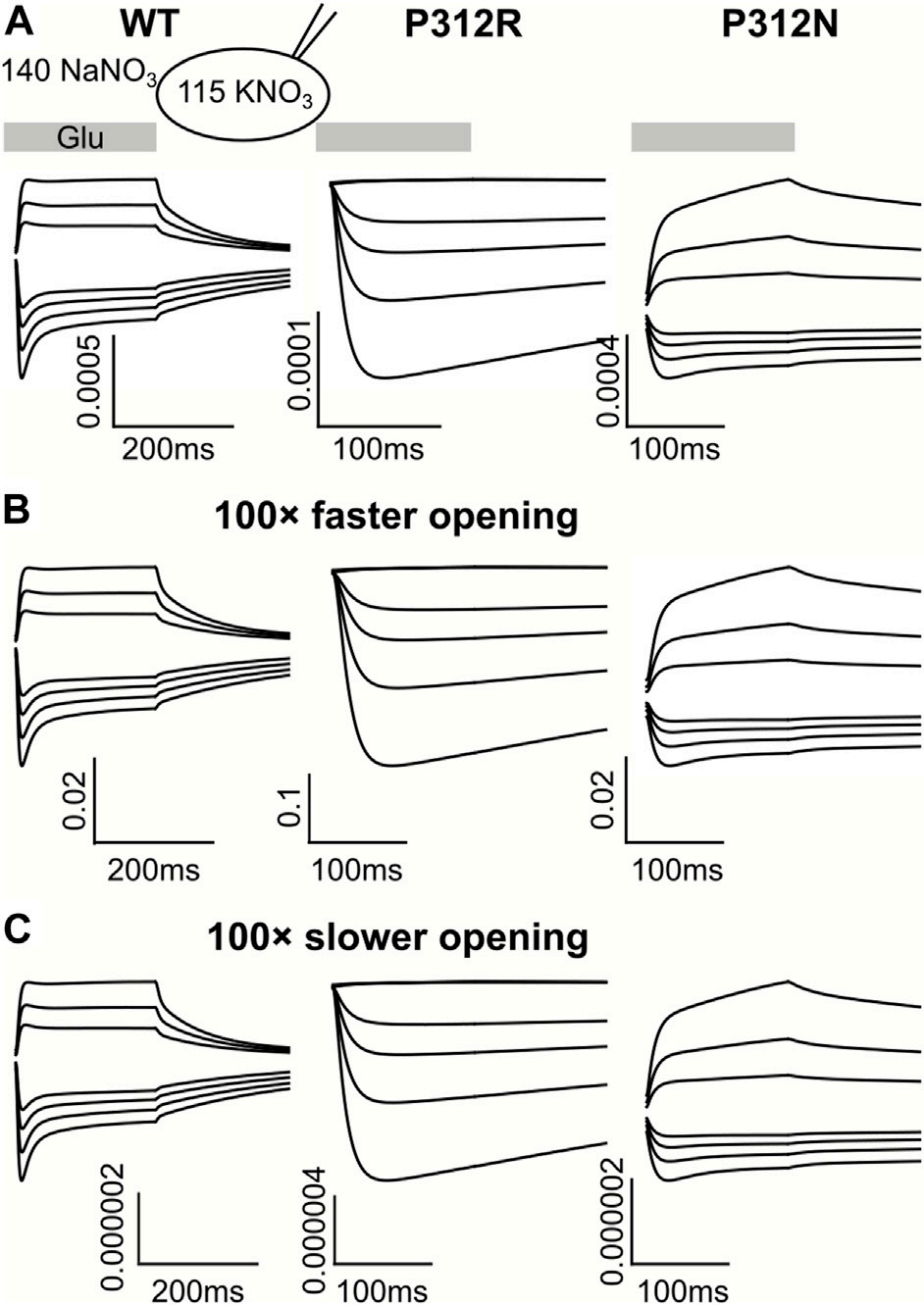
Kinetic modeling of WT and mutant EAAT4 anion currents does not require accurate knowledge of absolute anion channel open probabilities. Simulated anion currents for WT (left), P312R (middle) or P312N (right) EAAT4 with KNO_3_-based internal solution upon rapid application of L-glutamate as shown in Figure 6 **(A)**, or after increasing **(B)** or decreasing **(C)** anion channel opening rates by a factor of 100.

For some EAATs, absolute anion channel open probabilities can be obtained from the comparison of transport and anion currents, when absolute unitary transport rates are known (Fahlke et al., 2016; Kolen et al., 2020). Unfortunately, the absence of P312R and P312N EAAT4-mediated L-glutamate transport currents prevents application of this method for these particular mutations. Absolute open probabilities can therefore not be determined for these two mutants, and absolute current amplitudes cannot be analyzed. To test how our inability to quantify absolute open probabilities affects the outcome of modeling rates within the WT and mutant EAAT4 transport cycle, we compared predicted current time courses for WT and mutant EAAT4 upon variation of anion channel opening rates (Figure 11). Such changes resulted in dramatic alterations of absolute open probabilities, but left the time and voltage dependence of EAAT4 anion currents unchanged. We conclude that kinetic modelling of WT and mutant EAAT4 provides changes in transport rates of the transport cycle even in the absence of accurate values for absolute open probabilities. Most importantly, these results indicate that our modelling is unable to quantify anion channel opening rates.

## Discussion

We here combined heterologous expression, whole-cell recording and fast ionic substitution experiments with mathematical modelling to describe the molecular basis of glutamate transporter dysfunction in two inherited human diseases. Naturally occurring mutations that predict substitution of a conserved proline in the middle of the fifth transmembrane helix by arginine were found in patients with episodic ataxia type 6 (Jen et al., 2005), EAAT1) and in patients with epileptic encephalopathy (Epi4K Consortium, 2016; Guella et al., 2017), EAAT2). These mutations cause gain-of-function of the EAAT anion channel and reduce glutamate transport rates of both isoforms (Winter et al., 2012; Hotzy et al., 2013; Kovermann et al., 2022b). The substitution of proline by arginine might affect glutamate transporter function via long range electrostatic effects or by structural alterations that affect conformational changes underlying transporter function and anion channel opening. To distinguish between these two mechanisms of dysfunction we studied multiple substitutions of the homologous proline in EAAT4 (P312).

We chose EAAT4 because of its robust and well described anion currents in transfected mammalian cells (Melzer et al., 2003; Melzer et al., 2005; Kovermann et al., 2010; Leinenweber et al., 2011; Nothmann et al., 2011; Machtens et al., 2015). Unexpectedly, expression of most of the tested EAAT4 mutants resulted in only small anion currents (Figure 2). The majority of the mutations impaired surface-membrane insertion and resulted in predominant staining of intracellular membranes (Figure 4). For this reason, we could only study the functional consequences of neutral or positive. The similarity between P312K and P312R EAAT4 suggests that the substituted positive charge plays an important role in changing the voltage dependence of mutant EAAT4 currents. Substitutions of negative charges, i.e., P312E and P312D, were studied as additional test for electrostatic interactions, however, both mutations resulted in substantial impairment of intracellular trafficking (Figure 4), preventing functional analysis. P312N causes pronounced changes in function, indicating that not alone the charge, but also changes in transporter conformations contribute to the functional impairment of P312 mutations.

Colucci et al. recently reported the single-particle cryo-EM structure of the archaeal glutamate transporter homologue, Glt_Tk_, carrying the homologous P208R mutation (Colucci et al., 2023). They found the structure of the fifth transmembrane helix preserved in mutant Glt_Tk_ and the inserted arginine to project into the membrane. The authors suggested that interactions with the phospholipids might cause the observed changes in transport and anion channel activity. At present, our knowledge about the conformational changes resulting in anion channel opening and their changes by these structural alterations prevents a mechanistic explanation of the functional changes of mutant transporters. It will be interesting to see structures of mutant transporters carrying this mutation in additional conformations.

P312R and P312N cause distinct changes in the time and voltage dependences of macroscopic currents (Figure 1). P312R EAAT4 anion channels activate upon hyperpolarizing voltage steps. In contrast, P312N EAAT anion currents were time-independent and outwardly rectifying. Anion channel opening can only occur from certain states of the transport cycles, and changes in transitions within the transport cycle will therefore modify the voltage dependence of anion channel opening. To identify transitions that are altered by the studied point mutations we measured current responses to rapid changes in substrate concentration and fitted current responses to a 15-state kinetic scheme, to account for the complex transport stoichiometry and the dual function of EAAT4 as L-glutamate transporter and anion channel. The large number of states and the inability to directly measure the majority of individual rates make accurate and unambiguous determination of rate constants a complicated task. To account for this challenge, we studied WT and mutant transporters in an interative fitting procedure. After initial optimization, we tested whether fit parameters for mutant transporters differ from WT (Figure 8). If not, parameters were fixed to WT values in subsequent iterations, in order to reduce the number of variables. This procedure was three times repeated. We then tested the accuracy of our fitting results in an additional procedure, in which fit parameters were randomly modified, to define a range of values for each parameter that similarly well described the experimental data (Figure 9). This approach provided information about how well certain parameters are defined by the experimental values and our fitting procedure.

Optimization of reaction rates of the kinetic scheme demonstrated that P312R and P312N affected substrate association and dissociation, Na^+^/L-glutamate and K^+^-bound translocation (Table 1). Structures of mammalian EAATs and bacterial model proteins have been determined for multiple conformations, in inward and outward facing conformations (Yernool et al., 2004; Boudker et al., 2007; Reyes et al., 2009; Alleva et al., 2020) as well as in intermediate conformations (Verdon and Boudker, 2012; Chen et al., 2021). In all conformations, the residues homologous to P312, are not in close spatial proximity of any known structural determinant of Na^+^ or K^+^ association (Alleva et al., 2021). It thus remains unclear how P312 R/N affect cation binding/unbinding (Table 1).

Increased anion channel activity of P312R EAAT4 are caused by higher residence probabilities of existing conducting states as well as of T_CH_O, an open channel state accessible from outward facing *apo* state, and T_CH_K (Figure 10). Neither WT nor P312N transporters assume T_CH_O, suggesting that anion channel opening from outward-facing conformations is a direct consequence of the P312R mutation. One may imagine that increased occupation of anion channel modes that are outside the transport cycle might be the basis of the reduced transport rates. However, EAAT anion channels exhibit only very low open probabilities, and mutations that stimulate anion channel opening only minimally affected transport rates (Kolen et al., 2020). P312R and P312N reduce L-glutamate transport rates by impairing translocation (P312R) or substrate release (P312N, Table 1).

We recently employed voltage clamp fluorometry to study the functional consequences of the homologous proline by arginine mutation (P259R) on the human glutamate transporter EAAT3 (Hotzy et al., 2013). EAAT3—either carrying the P259R mutation or not–was optimized for voltage clamp fluorometry by inserting a cysteine at position 205 (M205C) and removing an endogenous cysteine at position 158 (C158S). After expression in *Xenopus* oocytes and fluorescent labeling, changes in fluorescence were evoked by voltage steps and studied for various external [Na^+^] or [L-glutamate]. A kinetic analysis of fluorescence signals suggested that P259R mainly affects Na^+^ binding to the glutamate-free transporter. Since two-electrode voltage clamp does not permit intracellular dialysis and thus greatly limits the analysis of translocation under distinct intracellular solution, it was impossible to identify changes in translocation or anion channel opening. Moreover, because of high anion background currents in oocytes, our earlier study was limited to the observation of fluorescence changes without analysis of anion currents. Such limitation prevented the identification of a major alteration in rate constants accessing open anion channel conformations from the *apo* state with this experimental system. In our present study, we also observed alterations of multiple Na^+^ binding steps to P312R EAAT4, however, the most pronounced change was enhanced unbinding of the third Na^+^, which binds after L-glutamate association and closure of HP2 (Guskov et al., 2016).

We here studied the functional consequences of disease-causing point mutations not in the affected glial EAAT isoforms, EAAT1 and EAAT2, but rather in the neuronal transporter EAAT4. This approach raises the question how our findings can help explaining the pathophysiology of episodic ataxia and epilepsy. Experiments with K^+^-based solutions identified increased activity of an anion channel that is accessible in the outward facing *apo* state as main reason of gain-of-anion channel function in P312R EAAT4. Since the mechanisms of K^+^ coupling are conserved in all EAATs, and since P290R EAAT1 and P289R EAAT2 share the unique K^+^ dependence with P312R EAAT4 (Winter et al., 2012), this result can certainly be transferred to the glial transporters, providing novel insights into the molecular pathophysiology of the diseases. Our modelling furthermore revealed changes in various transport transitions, demonstrating that P312R impairs the EAAT4 transport cycles in multiple transporter conformations. Again, the structural conservation between EAAT isoform suggest similar changes in mutant EAAT1 and EAAT2.

In recent years, an increasing number of genetic diseases has been identified that are caused by dysfunctional ion channels and were dubbed “channelopathies”. In many cases, the linkage of human diseases helped identification of cellular roles of the affected ion channels (Adrian and Bryant, 1974; Koch et al., 1992; Barhanin et al., 1996; Sanguinetti et al., 1996; Scholl et al., 2018; Schewe et al., 2019). Disease-associated mutations were often shown to result in defined changes in ion channel function (Cannon et al., 1991; Fahlke et al., 1997) and helped understanding the molecular basis of ion channel activation and conduction. Episodic ataxia type 6 was one of the first diseases caused by genetic dysfunction of transporters (Jen et al., 2005) and continues to illustrate the complexity of this group of diseases. The P290R mutation in EAAT1 causes ataxia not because of its impaired transport function, but rather by gain-of-anion channel function that induces Bergman glial cell apoptosis and cerebellar degeneration via increased Cl^−^ efflux (Kovermann et al., 2020). We here demonstrate that this disease-causing dysfunction is conferred by a hitherto unknown transporter state, an anion channel conformation that opens from a conformation, which usually prevents water and anion fluxes.

## Data Availability

The original contributions presented in the study are included in the article/Supplementary Material, further inquiries can be directed to the corresponding author.

## Supplementary material

SUPPLEMENTARY FIGURE S1 P312K and P312H cause distinct alterations in time, voltage and substrate dependences of EAAT4 anion currents.**(A)** Representative current recordings from HEK293T cells expressing P312K (left) or P312H (right) EAAT4 with a KNO_3_-based internal solution and a NaNO_3_-based external solution before and after application of 0.1 mM L-glutamate. **(B)** Voltage dependences of mean anion current amplitudes from cells expressing P312K (left) or P312H (right) EAAT4 obtained in cells intracellularly dialyzed with NaNO_3_-based solutions. Data are given as the means ± *C.I*.; *n* ≥ 4.

SUPPLEMENTARY FIGURE S2 P312R and P312N modify the glutamate dependence of EAAT4 anion currents **(A** )Representative current recordings from HEK293T cells expressing WT or mutant EAAT4 with a KNO_3_-based internal solution and a NaNO_3_-based external solution supplemented with various [L-glutamate] **(B)** Dose response curves of the L-glutamate dependence of WT or mutant EAAT current amplitudes. Data are given as the means ± *C.I*.; *n* ≥ 4, solid lines provide fits with Michaelis-Menten relationships. **(C)** Statistical analysis of apparent dissociation constants from fits shown in **(B)**.
