# Supplementary figures and images for: Apo state pore opening as functional basis of increased EAAT anion channel activity in episodic ataxia 6
Source: Front Physiol. 2023 Jul 19;14:1147216. doi: 10.3389/fphys.2023.1147216 (PMC10394623; doi:10.3389/fphys.2023.1147216)

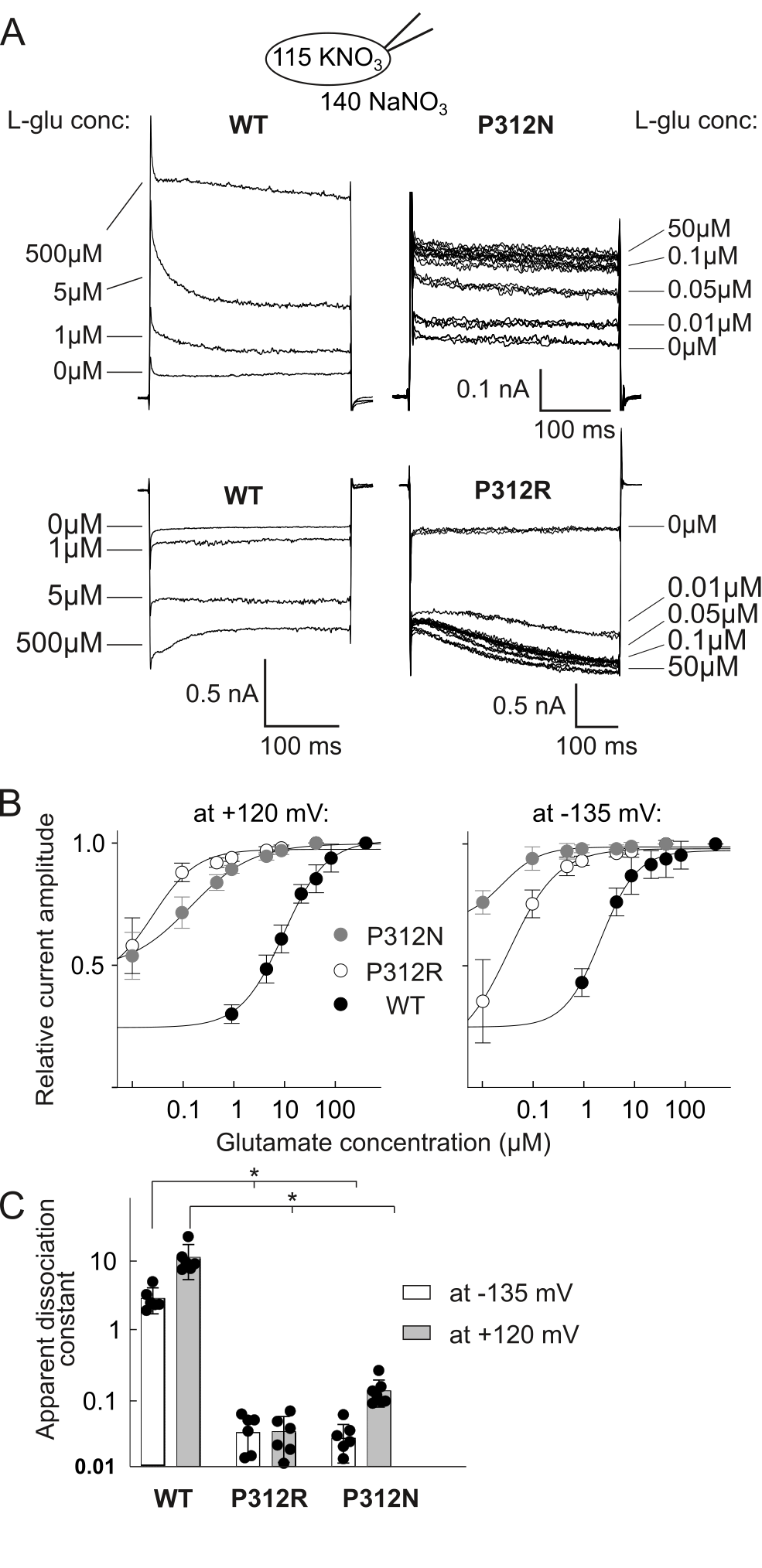

Supplement: Supplementary file 1 [file Image2.TIF]

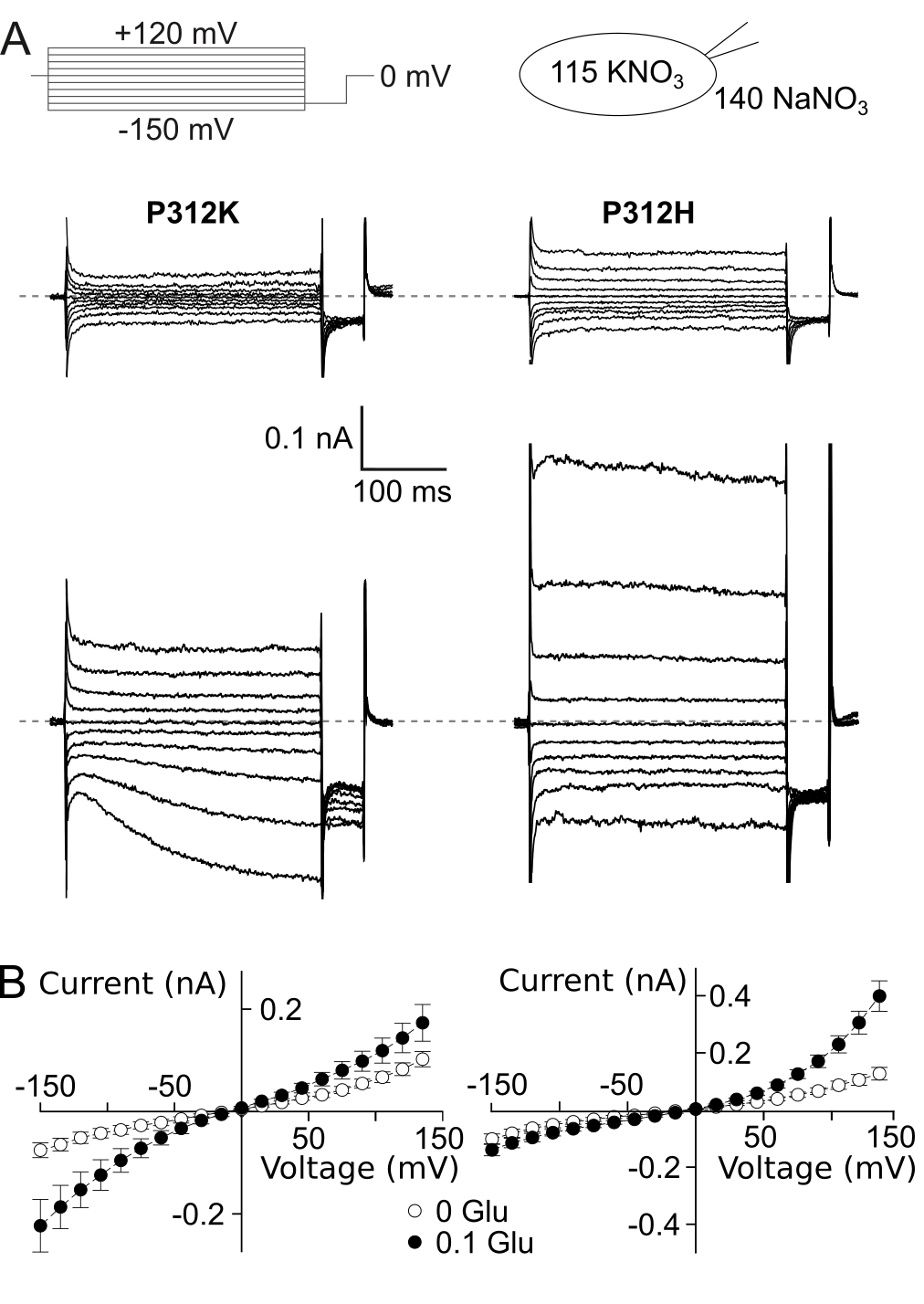

Supplement: Supplementary file 2 [file Image1.TIF]
